# Bioethics, children, and the environment

**DOI:** 10.1111/bioe.12386

**Published:** 2017-09-05

**Authors:** Timothy F. Murphy

**Keywords:** bioethics, children, environment, ethics, gay, lesbian, queer

## Abstract

Queer perspectives have typically emerged from sexual minorities as a way of repudiating flawed views of sexuality, mischaracterized relationships, and objectionable social treatment of people with atypical sexuality or gender expression. In this vein, one commentator offers a queer critique of the conceptualization of children in regard to their value for people's identities and relationships. According to this account, children are morally problematic given the values that make them desirable, their displacement of other beings and things entitled to moral protection, not to mention the damaging environmental effects that follow in the wake of population growth. Objectionable views of children are said even to have colonized the view of lesbian, gay, bisexual, and trans (LGBT) people who – with the enthusiastic endorsement of bioethics – increasingly turn to assisted reproductive treatments to have children. In the face of these outcomes, it is better – according to this account – that people reconsider their interest in children. This account is not, however, ultimately strong enough to override people's interest in having children, relative to the benefits they confer and relative to the benefits conferred on children themselves. It is certainly not strong enough to justify differential treatment of LGBT people in matters of assisted reproductive treatments. Environmental threats in the wake of population growth might be managed in ways other than devaluing children as such. Moreover, this account ultimately damages the interests of LGBT people in matters of access, equity, and children, which outcome is paradoxical, given the origins of queer perspectives as efforts to assert and defend the social interests of sexual and gender minorities.

## 1 INTRODUCTION

Cristina Richie advocates queering the field of bioethics as a way of improving current understandings of identity and moral responsibilities, not to mention reframing the ambitions of bioethics itself.^1^ Among other things, she imagines that queer bioethics will have the effect of reining in sociosexual values that are objectionable in themselves and that have harmful downstream effects on the environment. In the course of these accounts, Richie makes a fairly damning case against the value of children for people in general and for queer people in particular, arguing as she does that queer objectives and theory are incompatible with reproduction. In general, Richie presents children as a kind of social capital that is fundamentally compromised by the liberal framework of values that make them desirable, values that prioritize the future over the present. In particular, Richie argues that LGBT people are colonized by heteronormative values insofar as they interpret their own identities through values and relationships structured to the advantage of future children. Moreover, the ever increasing number of children – more or less uncritically enabled by researchers in fertility medicine and their cheerleaders in bioethics – puts at risk the welfare of human beings as well as – and just as importantly – flora, fauna, and natural objects that should themselves be sheltered under the umbrella of moral concern. Richie argues that any interest in children must be theorized in relation to “all creatures sharing our planet”, and *not only living organisms*, because waterways, “sand counties”, and other non‐living natural features have moral standing too, as legitimate objects of concern about their treatment and fate. In this way, she makes the case that bioethics should return to its origins in concerns of exactly this kind. On a bionatural account like this, it may not be inherently irresponsible to have a child, but for the most part Richie characterizes people's interest in children as evidence of and complicity with a raft of values objectionable in themselves and in their environment‐endangering effects. The theory and practice of having children is, according to this account, fundamentally damaging, and – allegedly – queer perspectives are poised to identify and help correct reproductive values, as they also do other work to elevate bioethics to its full potential as an instrument of critique and evaluation.

This account of queer bioethics ultimately works to the disadvantage of LGBT people, which is a very paradoxical outcome for perspectives originally having their origins with these sexual minorities. Richie does in this current project what she has done elsewhere: burden sexual minorities with social responsibilities without parallel for others.^2^ She also undervalues the nature and meaning of barriers that obstruct LGBT people having children. If social values must be reconfigured for moral reasons bearing on the environment, it is unclear that LGBT people have any more responsibility in this regard than anyone else. That conclusion has all the more force if we decline to accept an interpretation of non‐living things as having moral priority over the interests of human beings and other sentient creatures.

## 2 QUEERING BIOETHICS

Commentators characterize “queer” perspectives in various ways, but almost always as a corrective to certain sociosexual orthodoxies of one kind or another.^3^ For her part, Richie characterizes ‘queer’ as – among other things – a political stance which challenges liberal values which are said to be: production, conformity, sameness, and homogenization. Richie deploys “queer” as a critique of normativity, by which she means the reigning “figment of what a person should be/is,” in all the metaphysical and moral implications of that identity. More ambitiously expressed, she says that queerness means that “normativity of all kinds is critiqued, rejected, and abandoned.” She characterizes “queerness” in contradistinction not only to heternormativity outright – in its domineering views of relationships as structured around a certain kind of opposite‐sex relationship – but also in regard to the heternormativity she says has colonized the self‐understanding of LGBT people. As she sees matters, then, queer bioethics is a kind of oppositional defiance, without *a priori* commitments to any particular normativity and/or social categories. She says, for example, that queer bioethics “is a field that fundamentally opposes categorizations, favoring pastiche to principle”. In effect, she represents “queer” as skeptical thought itself, a view from nowhere, as it were, and therefore exempt from certain expectations of consistency.

This characterization of queerness draws deeply from the intellectual playbook of modernism itself, which has always involved deep questioning, irreverent attitudes toward life, deep skepticism toward official structures of society, and unwillingness to pay reflexive homage to authority across the board.^4^ Modernism takes very little for granted by way of the alleged nature of things, the socially asserted moral order, presumptive ways of knowing, or even standard accounts of the beautiful. Even when presented as a stand‐alone venture, queer bioethics has precedents in metaphysics, morality, epistemology, and aesthetics, all of these precedents sharing skepticism of received orthodoxies.

Richie says variously that queer perspectives are essential to ethical evaluations of children if not bioethics itself, insofar as she sees them functioning as tools for overthrowing the status quo across the board. She would train their efforts on concepts undergirding identity, medical relationships, systems of authority, not to mention the “distasteful” axes of power involved in capitalism, but above all else: reproductive normativity. She says that this normativity must be rejected “in order to maintain a radical stand that exemplifies Queerness itself.” As a way into showing the failings of bioethics in these domains, Richie takes pains to distinguish queer bioethics from its historical forerunner, LGBT bioethics, portraying queer bioethics as a corrective to LGBT bioethics which she characterizes as in thrall to heteronormativity among other failures. LGBT bioethics is merely reformist, while queer bioethics is characterized as revolutionary. It comes as no surprise, then, that Richie objects to LGBT bioethics assigning central moral significance to children, especially to the extent LGBT people conceptualize children, want them, have them, and rear them just as anyone else would. On Richie's view, any analysis that simply works to extend the right of LGBT people to have children under those terms misses more ultimately important questions about the ethics of having children in the first place.

Some commentators take a more nuanced view of children in relation to LGBT understandings of self and relationships,^5^ but as Richie sees matters, working to open the prospects for LGBT people to have children counts as complicity with the moral lapses and environmental harms that are inextricably rooted in heteronormative parenting. (Other commentators have pointed out the changes in identity involved as sexual and gender minorities seek out fertility services, as well as certain moral complicities connected to engagement with the practices of global fertility medicine.)^6^ Any defense of LGBT people having children – via adoption, assisted reproductive treatments (ARTs), and even with imaginable technologies on the horizon – can only leave LGBT people and their defenders in bioethics in the throes of objectionable liberal values, with their lives and families contributing to the wrongful devaluation of the environment and its degradation too.

To show how much work remains to be done by queer bioethics, Richie has singled out my work as instructive in the limitations of LGBT bioethics. For example, she describes my work defending access and equity for LGBT people in regard to ARTs as having “an affinity with” the pronatalist work of the United States Conference of Catholic Bishops. The standard meaning of “affinity” is: “a sympathy esp. as marked by a community of interests.”^7^ I can't explain why Richie attributes to my *writing* a feeling of closeness to the views of others, when affinities properly belong to *people,* not texts. For her purposes, “homology” would have been a better term because that means “having the same relative position, value, or structure.”^8^ That seems to be the point she is making, that my work has homologies with the perspectives of the Catholic bishops, but even then the point is forced, especially since the point of comparison is compressed into sloganeering pathos: “*Think of the children*”.

The U.S. Catholic Conference of Bishops works on behalf of a church that as a matter of theory and practice works against the status equality of LGBT people. For example, let's recall that Church's 1975 and 1986 declarations that homosexual act are “deprived of their essential and indispensable finality,” that they are “intrinsically disordered,” and that they may in no case be approved of.^9^ On the basis of this metaphysical account, the Catholic Church recommends excluding people who engage in this kind of sexuality from various social goods.^10^ For example, the Catholic Church objects to same‐sex marriage and – when it comes to having children – ARTs for anyone, the cloning of human beings, and synthetic gametes. These positions generally follow from the view that the conception of children should occur as an effect of sexual intercourse between a man and woman who are married to one another and committed to a certain interpretation of responsible parenthood. The leadership of this church has said, among other things, “Marriage and conjugal love are by their nature ordained toward the procreation and education of children. Children are really the supreme gift of marriage and contribute in the highest degree to their parents’ welfare.”^11^

Am I in bed with Catholic bishops? Not in a way that would make any of us very happy, even speaking only conceptually. When sex between men and sex between women was a crime in most of the United States, I defended the morality integrity of that kind of sex.^12^ I have defended the right of same‐sex couples to marry as a matter of law, and I have even criticized the Ontario court decision legalizing same‐sex marriage in that country for not offering a robust enough defense of that kind of marriage.^13^ I have defended access and equity in regard to fertility medicine for gay men and lesbians without regard to any requirement of marriage.^14^ I have defended the right of gay and lesbian people – as single people or couples – to rely on any safe and effective method for cloning human beings that might come along.^15^ I have defended the right of people to take steps to have gay and lesbian children if that were possible through certain kinds of prenatal interventions.^16^ I have defended the interests of transwomen in uterus transplantation for gestation.^17^ I have defended the prospect of same‐sex couples relying on synthetic gametes – should they to become available – to conceive children as couples.^18^

Nothing in this work depends on the idea that sexual relationships not open to conception are fundamentally disordered. Nothing in this work depends on the idea that the very nature of adult sexual relationships is teleologically ordained to produce children. Nothing in this work requires that LGBT people have children in order to assign moral significance to themselves or their relationships, whether coupled or not. Nothing in this work depends on the idea that sexual relationships between men or between women are subordinate in their value to sexual relationships between men and women. Nothing in this work requires that identities be understood as valuable only in relation to children. When it comes to children, my work has been concerned with – above all – defending access and equity to social goods. Nothing in this work requires that people enter into monogamous, life‐long relationships as a condition of “eligibility” for children. I have made no judgment about whether individual gay men, lesbian, bisexual people, or trans people ought to have children or whether they ought to do so as a class, but I have worked to show that there are no meaningful moral obstacles to their doing so in the context of relationships and identities that may differ from social expectations.

All things considered, I have been no friend to the formal teachings of Catholicism. Perhaps the rationale for Riche's claim about my work is the idea that *any* moral argument that favors the having of children in one way or another amounts to the ‘pronatalism’ expressed by theorists of Catholicism. If so, the label doesn't’ mean very much descriptively and amounts to almost nothing as a critique. At this point, however, let me take up directly the question of whether the interest in having children can survive Richie's critique, especially for LGBT people.

## 3 THE MEANING OF CHILDREN

Central to Richie's analysis is the repudiation of children as fundaments in the value of human life. She declares that they have a warping effect on identities and relationships, deflect attention from the present to the future, and that they undercut an ethic of bionatural ecology. “*Don't think of the children!*” might be her rallying cry. In fact, *nowhere* in her analyses does she characterize children as having value of any kind.^19^ She does say: “While certainly reproduction can be a significant part of some people's lives, Queer bioethics offers a competing discourse to the one that assumes reproduction is a focal part of a person's life plan.” To say that children are a significant part of a life is not the same as endorsing that role. She says, in fact, that “it should not be assumed that biological parenthood has value for all people, *or any people*, inclusive of Queers” [emphasis added]. By way of emphatic punctuation to that point she also says that since “heteroreproduction is repudiated by Queer studies, bioethical banter need not waste undue time promoting reproduction.”

This is not the place for a full‐frontal engagement with the ethics of having children. I will note, however, that defenses of having children are at least as robust as the commentary offered against having children.^20^ For example, while I do not believe that Richard Kraut succeeds in his argument that people are generally obliged to have children generation after generation, indefinitely into the far reaches of time, I accept that his argument certain gives people moral permission to have children.^21^ Children are valuable in a variety of ways: to people for the rewards that inhere in parental identity, for the goods available in parent‐child relationships, and for the benefits life confers on children themselves. I take these goods to be prima facie justification enough for having children as a matter of principle and, I submit, justification enough for efforts in bioethics to help people with obstacles to having children. At the very least, I do not accept that locating children within liberal and/or capitalistic and/or religious interpretations by itself undercuts their value as desirable. Neither does imagining the benefits of a life without children for some people establish the valuelessness of children in general.

Kraut's analysis also deserves mention another way, in its rejection of any version of the idea that some supravrening good confers value or is the reason by which anything is of value. He argues, for example, that no inherent good is expressed or achieved by there being a particular kind of *living thing* in existence, or in its continuance for that matter. No inhering good is expressed or achieved by there being a particular kind of *non‐living thing* in existence or in its continuance. What this view means is, in brief, that living things (flora, fauna) and natural features (waterways) have no moral interest over and above human interest in them. They can still be protected and preserved, should human beings decide to do so, but there is no good per se in them that requires their protection and perpetuation. Although I won't develop the particulars of Kraut's argument here, I will say that an account like this would undercut the bionaturalism that Richie has put forward. On this account, no flower or plant or waterway – or any possible collection of such things – can by virtue of its existence have a claim to continuing existence or be protected by a rule of non‐interference. This is all by way of saying that Richie's account of the value of living and non‐living things – enfolded under the protection of queer bioethics – is more asserted than defended against competing accounts of the ways in which things have normative value and/or moral status.

## 4 THE STATUS OF LGBT PEOPLE

Let me say a bit more here about children in relation to LGBT people. Some LGBT people have children through conception in opposite‐sex relationships, through adoption, through foster care, or through ARTs. It is an understatement to say that they have done so without endorsement from any major social authority. It is also an understatement to say that they have done so in sometimes inventive ways, compared to cultural expectations.^22^ This is not either the place for a full‐fledged defense of the right of LGBT people to have children, but I think it is more than fair to say that LGBT people have faced socially chosen obstacles without parallel for others, and – I submit – for no good moral reason. If I may put an only slightly provocatively spin on Richie's account: any obstacles in the way of LGBT people having children have ironically protected them from the parent trap! That is, those obstacles to having children have protected many LGBT people from succumbing to heteronormative reproduction. Seen from this perspective, LGBT people are maybe now worse off than before in relation to heteronormativity, because many more now live in societies giving them more opportunities – and hence more pressure – to have children, whereas they would have in the past been “protected” from this morally compromised opportunity.

To be sure, the obstacles to parenthood by LGBT people are diminishing in force in significant swaths of the world, though not all. For example, prominent medical organizations have defended the entitlement of gay, lesbian, and transmen and women to ARTs.^23^ Whether that right is fully observed in practice is a different question, of course, but these professional organizations have asserted the status equality of gay, lesbian, and transpeople in regard to access and equity in such treatments.^24^ For her part, Richie is unconcerned about matters of access and equity in regard to children. On the contrary, she objects almost on principle to the place of children in the aspirations of LGBT people, at least insofar as they ought to be the front line of queer interests. Not only does she see LGBT people as colonized by heteronormativity in regard to children – which effect she calls “homonormativity”– she has also offered policy recommendations that would obstruct LGBT access to ARTs and other healthcare interventions.

For example, Richie has argued against government and insurance subsidy for ARTs for same‐sex couples.^25^ She justifies this exclusion by arguing that healthcare ought to confine itself to the treatment of disorders properly speaking, and two men or two women wanting to have a child suffer no pathology that obstructs conception and/or gestation of a child. This kind of argument has intellectual precedents in, for example, Leon Kass's strict interpretation of medicine as focused on “health”, but the argument is vulnerable to criticism especially from the countervailing view that healthcare may legitimately aim at “well‐being” in an expansive sense and not just “health” in some restrictive sense alone.^26^ From this view, ARTs contribute to the well‐being of same‐sex couples even if they do not involve treating an underlying disease or disorder.

Even apart from this flimsy account of eligibility for fertility interventions, Richie's justification for turning away subsidized ARTs for gay and lesbian people is curiously an adverse preference, something chosen because a better option is not available.^27^ She raises it only after dismissing another option, one that would have affected opposite‐sex and same‐sex couples equally. She says that “while a moratorium on all ARTs would be the most ecologically sound decision … it is *unlikely* that established fertility procedures or treatments would be effectively ‘banned’ until global CO_2_ emissions stablise.”^28^ In other words, there is a better way to reduce the damaging effects of children: close off all ARTs, but Richie offers exactly *no defense* of this “most ecologically sound decision”. Instead, she shifts the burden for carbon emissions to same‐sex couples, on the grounds that withdrawing any tax or insurance subsidy for lesbian and gay people looking for clinical help in having a child would be for some unspecified reason *likelier*. I suppose it would be likelier if what is meant is that the straight beneficiaries of subsidized ARTs would be unwilling to see that perk taken away; as a political matter that “best option” would be dead on arrival. But apparently Richie foresees sees no comparable political backlash if subsidized ARTS disappeared for LGBT people. In other words, the perceived political powerlessness of LGBT people counts as part of the rationale in favor of her exclusion of LGBT people from tax or insurance subsidy for ARTs!

Neither would Richie guarantee body modifications to transpeople wanting them as a means of gender expression, at least in principle. Keeping the environment in mind, she says “many body projects that demand medical intervention are at odds with Queer environmental bioethics and are ecologically unsustainable.” In other words, all healthcare services have a carbon footprint, and the effect of these interventions will have to be evaluated relative to their impact that way. A transwoman might want, for example, hair removal, eyebrow reduction, nose and earlobe resizing, Adam's apple size reduction, hormone treatment, excision of penis and testes, reposition of the urethra, among other body modifications. The day might come, too, when transwomen might want uterus transplants in order to experience gestation to the extent possible, in other words, for *exactly* the same reason that cisgender women want uterus transplants. As these examples make clear, in their totality these interventions would not be without meaningful environmental effects.

As a matter of principle, Richie is prepared to close these options off, were they implicated as an unacceptable environmental burden, but she is unimpressed with the damage this denial of services might do to transpeople: “This internal contradiction reifies the liminality, fluidity, and flux of Queer bioethics, which defies uniformity in description or application.” Whatever else this word salad means, it means Richie is excusing herself from responsibility for an outcome that could work against the interests of transpeople. Yet, if we are going to begin assessing body modifications as threats to the environment, it is not clear why a small fraction of people having body modifications as a way of managing gender dysphoria should be closed out of those modifications, compared to the vast number of cisgender people having body modifications in the name of their own gender expression, modifications such as fat removal, fat redistribution, body sculpting of all kinds, genital enhancements, dermabrasion, tattooing, tattoo removal, and all the other body modifications sought as a matter of gender expression (bringing one's body into line with one's gender ideal). In their totality, the body modifications sought by vast numbers of cisgender people will have more environmental impact than the body modifications sought by the always meaningfully smaller number of transpeople.

The review here shows exactly how queer bioethics – in the way Richie formulates it – loses sight of LGBT people in their particularity. Its generic characterization as *skepticism as such* overrides social circumstances important to lesbian, gay, bisexual, and transpeople, adults or adolescents. To be clear, I am not saying that LGBT people never misapprehend the meaning of children or that LGBT people have no environmental responsibilities, but I am asking why LGBT people as a class must be theorized as ‘liberated’ from interest in children as part of their environmental responsibilities and why LGBT people should have any more responsibilities toward the environment than anyone else. Gay ‘theorists’ such as Lee Edelman – whom Richie invokes – may interpret children as utterly beside the point of queer life, but that view is itself an interpretation, a normative “figment” of what a person is or ought to be.^29^ It's not even clear that the specific proposals put forward by Richie would make much difference in regard to the protection of the environment, namely withdrawing government and insurance subsidy from same‐sex couples looking for help having children via ARTS and/or shutting down body modifications for transpeople. In general, it makes sense to put solutions where the problems are, and these proposals don't do that. What is altogether lacking in Richie's analysis is any mechanism for allocating responsibility for environmental threats according to some metric of responsibility and a mechanism for prioritizing one constraint on environmental damage over another. Without methods for establishing this kind of responsibility and priorities, the choices Richie champions are not only unprincipled, they are almost random, not to mention punitive to LGBT people.

## 5 CONCLUSIONS

Against the background of a bionaturalist ethic giving moral status to non‐living things and living things alike, Richie has applied a version of modernist skepticism to the valuation of children. In general, she characterizes the desire for children as morally suspect in itself and damaging in its effects. By Richie's account, children are metaphysically unnecessary in a good life, are in many ways desired for dubious reasons, and – collectively – are deeply damaging in their effects on the environment. Wanting and having children is impossible without complicity in objectionable moral values and social practices, and the interests of LGBT people in having children are not exempt from this indictment.

To Richie's dismay, bioethics has pretty much only ever worked to enlarge the prospects for adults having children. LGBT people have benefited from that effort with a degree of success almost without parallel in the field. Robust defenses of LGBT people as parents, their entitlement to existing ARTs, and entitlement to techniques of assisted reproduction looming on the horizon are the rule in bioethics; opinion to the contrary is no more prevalent than it is convincing.^30^ Richie wants to intervene against the enthusiasm for ever‐expanding options for having children, but this intervention undoes some of the gains most important to men who have sexual interest in men, women who have sexual interest in women, people whose gender expression hits social tripwires, and people whose polyvalent sexual and romantic identities do not map easily onto socially authorized roles. Accordingly, LGBT people become a casuality of Richie's bionaturalism. If we take seriously her principled opposition to normativities “of any kind”. LGBT identities have no special status, any more than do heteronormative “figments” of what it is to be a person, especially in relation to children. In the face of looming environmental disaster, we are only – all of us – unindividuated entities. People's sexual orientations and gender identities are eclipsed in significance in the shadow of present and looming ecological damage. The integrity and value of LGBT identities are simply casualties of an underlying shift toward bionaturalism, in which *no* future can be more important than the present, not if getting there involves the self‐deceiving and morally objectionable values attached to children or involves present harms tolerated in the name of benefit to future people. Thus is to be understood Richie's commitment to immanence: “Queer bioethics is now.” Immured in the present, Richie feels free to overlook the historical and hard‐won social gains represented by defending access and equity for LGBT people in having children. That way, she is free to treat questions about access and equity in regard to children as almost entirely beside the point, as so much “banter.”

Even so, the generations to come remain of moral interest, perhaps not as a matter of moral obligation properly speaking, but certainly as a matter of beneficence toward others yet to live. Richie might have pressed a case for environmentalism without diminishing the value of children per se and without also singling out children as especially problematic for LGBT people. For example, she might have conferred on each and every person some measure of responsibility for the morally relevant global environment. If it really is necessary to lower the total birth rate to preserve the environment, not just for prudential reasons but for moral reasons, Richie could have examined, for example, ways to ration children by some equitable mechanism, and we could have a searching discussion about what form that would take: “Just exactly what number of people is compatible with benchmark environmental goals? Given that the interest in children probably exceeds the total allowable number, how should they be apportioned?” Or, to go another way, Richie might have proposed social incentives for refraining from having children. These approaches shift the responsibility for the welfare of the global environment across all people, perhaps even giving each person some measure of responsibility for deciding how many human beings come into existence. Instead of trying to apportion responsibility for the environment in any equitable way, Richie opts to deconstruct the value of future lives, so much so that she in fact leaves open the prospect of an *intentionally failed future as liberating*.^31^ Rather than assigning responsibility for the environment to all, she opts to defend certain choke points against more people coming into existence: denying government and insurance support for fertility medicine for same‐sex couples, leaving synthetic gametes unstudied in the laboratory, and possibly limiting interventions such as uterus transplants that could confer certain procreative powers on transmen and women.

Taken together, these recommendations would do very little to stem the overall tide of population growth, because of the fractional numbers of people involved. Even if the numbers were more significant, it is still not clear why LGBT people should be required to assume any responsibility for the future not equally shared by all other parties. All things considered, one might make exactly the contrary case: that LGBT people ought to have priority on any list of people entitled to have children, even if there were a diminution in the morally allowable number of children in the future. In view of the cultural, religious, moral, and legal obstacles that impede LGBT people having children one can imagine such an argument – on the grounds of compensatory justice – that LGBT people ought to have some priority over others in having children, should there ever be a general rationing mechanism. At the very least, this question would have to be answered: Why should people who – as a class – have never faced socially imposed obstacles to having children be presumed entitled to first consideration for children ahead of people who – as a class – have faced socially imposed and morally unjustifiable obstacles to having children?

Richie points to queer bioethics as a way to elevate bioethics to its full potential as an instrument of critique and evaluation. That is, queer bioethics is not just a subdomain of bioethics focused on a particular group of people. Queer bioethics is or ought to be bioethics itself, by exhibiting a thorough‐going Cartesian skepticism toward all received knowledge and its instruments. In this exposition of queer bioethics, the interests of LGBT people in having children come under frontal assault on the grounds that they are unnecessary to a good life, that they are desirable only in morally compromised ways, and that they do morally significant environmental damage. This queering of bioethics is of little help to LGBT people because it treats the value of LGBT lives at a deep discount in matters of access and equity. It is, sad to say, in many ways homologous with commentary that intentionally works against status equality for LGBT people.

## Footnotes

1
Richie, C. (2016). Lessons from queer bioethics: A reply to Timothy F. Murphy. *Bioethics*, *30*, 365–371. Unless otherwise specified, citations from Richie are from this article. 10.1111/bioe.1224626833492

2
Murphy, T. F. (2015). Against withdrawing government and insurance subsidies from ARTs from fertile people, with special reference to lesbian and gay individuals. *Journal of Medical Ethics, 41*, 388–390. 10.1136/medethics-2014-10250725657262

3
See Wahlert, L., & Fiester, A. (2012). Queer bioethics: Why its time has come. *Bioethics, 26*, ii–iv; Wahlert, L. (2015). Mapping queer bioethics: Space, place, and locality. *Journal of Homosexuality*, epublished Dec. 7.

4
Hughes, R. (2006). *Goya* (pp. 10–11). New York: Knopf.

5
Mamo, L. (2013). Queering the fertility clinic*. Journal of Medical Humanities*, *34*, 227–239. 10.1007/s10912-013-9210-323463357

6
Mamo, L. (2007). *Queering reproduction: Achieving pregnancy in the age of technoscience*. Durham: Duke University Press.

7
Babcock Gove, P. (Ed.) (1993). W*ebster's Third New International Dictionary* (p. 35). Springfield, MA: Merriam‐Webster.

8
Simpson, J. A., Weiner, E. S. C. (Eds.) (1988). Homologous. In *Oxford English dictionary* (2nd ed., vol. VI, p. 418). Oxford: Clarendon Press.

9
Congregation for the Doctrine of the Faith. (1975). Persona humana: Declaration on certain questions concerning sexual ethics. Retrieved from (http://www.vatican.va/roman_curia/congregations/cfaith/documents/rc_con_cfaith_doc_19751229_persona-humana_en.html); Congregation for the Doctrine of the Faith. (1986). Letter to the Bishops of the Catholic Church on the Pastoral Care of Homosexual Persons. Oct 1. Retrieved from (http://www.vatican.va/roman_curia/congregations/cfaith/documents/rc_con_cfaith_doc_19861001_homosexual-persons_en.html).

10
For example, that Church raises a bar against ordination to the priesthood for “those who practice homosexuality, present deep‐seated homosexual tendencies or support the so‐called ‘gay culture.’” See Congregation for Catholic Education. (2005). Instruction concerning the Criteria for the Discernment of Vocations with regard to Persons with Homosexual Tendencies in View of their Admission to the Seminary and Holy Orders. Nov. 4. Retrieved from http://www.vatican.va/roman_curia/congregations/ccatheduc/documents/rc_con_ccatheduc_doc_20051104_istruzione_en.html) This view was reaffirmed by the Congregation for the Clergy. (2016) The Gift of the Priestly Vocation.” Dec. 8. Retrieved from http://www.clerus.va/content/dam/clerus/Ratio%20Fundamentalis/The%20Gift%20of%20the%20Priestly%20Vocation.pdf.

11
Paul VI. (1968). *Humanae vitae* July 25. Retrieved from http://w2.vatican.va/content/paul-vi/en/encyclicals/documents/hf_p-vi_enc_25071968_humanae-vitae.html

12
Murphy, T. F. (1994). Homosex/Ethics. In T. F. Murphy (Ed.), *Gay ethics: Controversies in outing, civil rights, and sexual science* (pp. 9–25). Binghamton, N.Y.: Haworth Press

13
Murphy, T. F. (2011). Same‐sex marriage: Not a threat to marriage or children. *Journal of Social Philosophy*, *42*, 288–304.

14
Murphy, T. F. (2015). Against withdrawing government and insurance subsidies for ARTs from fertile people, with special reference to lesbian and gay individuals. *Journal of Medical Ethics 41*, 388–390; Murphy, T. F. (2016). LGBT people and the work ahead in Bioethics. *Bioethics* 29, ii–v. 10.1136/medethics-2014-10250725657262

15
Murphy, T. F. (1999). Entitlement to cloning. *Cambridge Quarterly of Healthcare Ethics, 7*, 364–368. 10475694

16
Murphy, T. F. (1997). *Gay science: The ethics of sexual orientation research*. New York: Columbia University Press.

17
Murphy, T. F. (2015). Assisted gestation and transgender women. *Bioethics*, *29*, 389–397. 10.1111/bioe.1213225522123

18
Murphy, T. F. (2014). The meaning of synthetic gametes for gay and lesbian people, and bioethics too. *Journal of Medical Ethics*, *40*, 762–765. 10.1136/medethics-2013-10169924489106

19
C. Richie gives a robust defense of childlessness in, Richie, C. (2013). Disrupting the meaning of marriage. *Theology and Sexuality*, *19*, 123–142.

20
For example, see Overall, C. (2012). *Why have children? The ethical debate*. Cambridge: The MIT Press.

21
Murphy, T. F. (2016). What justifies a future with humans in it? *Bioethics*, *30*, 751–758. 10.1111/bioe.1229027667120

22
Luce, J. (2010). *Beyond expectation: Lesbian/bi/queer women and assisted conception*. Toronto: University of Toronto Press.

23
Ethics Committee. (2015). Access to fertility services by transgender persons: An Ethics Committee report. *Fertility and Sterility, 104*, 1111–1115. See also Committee on Healthcare for Underserved Women. (2011). Health care for transgender individuals. *Committee Opinion Number 512* Retrieved from https://www.acog.org/Resources-And-Publications/Committee-Opinions/Committee-on-Health-Care-for-Underserved-Women/Health-Care-for-Transgender-Individuals 10.1016/j.fertnstert.2015.08.02126363388

24
Roth, A. (2016). So this lesbian couple walks into a fertility clinic. *American Philosophical Association Newsletter on Philosophy and Medicine*, *15*, 6–12.

25
Richie, C. (2014). What would an environmentally sustainable reproductive technology industry look like? *Journal of Medical Ethics*, E‐published July 24. 10.1136/medethics-2013-10171625060852

26
Kass, L. (1988). *Toward a More Natural Science*. New York: Basic Books.

27
Murphy, *op. cit*. note 17.

28
Emphasis added. Richie, *op. cit*. note 1.

29
Edelman, L. (2004). *No future: Queer theory and the death instinct*. Durham: Duke University Press.

30
Among the naysayers to LGBT people as parents, except under very unusual circumstances is: Somerville, M. (2004). *The ethical canary: Science, society, and the human spirit* (pp. 47 ff). Montreal: McGill‐Queen's University Press.

31
Emphasis added. Richie, *op. cit*. note 1, p. 371. Richie counts herself among those who “prefer extinction to a carbon legacy.” Just to be clear: her phrase “carbon legacy” stands in for children. Richie is free, of course, to invoke the language she wishes to characterize her choice against having children as her “extinction”, but except as a poetical indulgence “extinction” is a poor term to refer to the death of a childless person. I would myself find it very entertaining to read an obituary that said something like this: “Samantha Jones went extinct on June 1, 2016, leaving no carbon legacies behind.” As a matter of standard usage, “extinction” refers to the death of a class of beings in kind. Only if she were the last human being alive would anyone's death constitute extinction properly speaking.

